# Glucosamine and Silibinin Alter Cartilage Homeostasis through Glycosylation and Cellular Stresses in Human Chondrocyte Cells

**DOI:** 10.3390/ijms25094905

**Published:** 2024-04-30

**Authors:** Yu-Pao Hsu, Tsung-Hsi Huang, Shu-Ting Liu, Shih-Ming Huang, Yi-Chou Chen, Chia-Chun Wu

**Affiliations:** 1 Department of Orthopedics, Taoyuan General Hospital, Ministry of Health and Welfare, Taoyuan City 330, Taiwan; dryphsu@gmail.com (Y.-P.H.); fossnack@gmail.com (T.-H.H.); 2 Department of Biochemistry, National Defense Medical Center, Taipei City 114, Taiwan; shuting0719@gmail.com (S.-T.L.); shihming@ndmctsgh.edu.tw (S.-M.H.); 3 Graduate Institute of Medical Sciences, National Defense Medical Center, Taipei City 114, Taiwan; 4 Department of Orthopedics, Tri-Service General Hospital, National Defense Medical Center, Taipei City 114, Taiwan; 5 Institute of Preventive Medicine, National Defense Medical Center, New Taipei City 237, Taiwan

**Keywords:** osteoarthritis, cartilage homeostasis, glycosylation, endoplasmic reticulum stress

## Abstract

Osteoarthritis is more prevalent than any other form of arthritis and is characterized by the progressive mechanical deterioration of joints. Glucosamine, an amino monosaccharide, has been used for over fifty years as a dietary supplement to alleviate osteoarthritis-related discomfort. Silibinin, extracted from milk thistle, modifies the degree of glycosylation of target proteins, making it an essential component in the treatment of various diseases. In this study, we aimed to investigate the functional roles of glucosamine and silibinin in cartilage homeostasis using the TC28a2 cell line. Western blots showed that glucosamine suppressed the N-glycosylation of the gp130, EGFR, and N-cadherin proteins. Furthermore, both glucosamine and silibinin differentially decreased and increased target proteins such as gp130, Snail, and KLF4 in TC28a2 cells. We observed that both compounds dose-dependently induced the proliferation of TC28a2 cells. Our MitoSOX and DCFH-DA dye data showed that 1 µM glucosamine suppressed mitochondrial reactive oxygen species (ROS) generation and induced cytosol ROS generation, whereas silibinin induced both mitochondrial and cytosol ROS generation in TC28a2 cells. Our JC-1 data showed that glucosamine increased red aggregates, resulting in an increase in the red/green fluorescence intensity ratio, while all the tested silibinin concentrations increased the green monomers, resulting in decreases in the red/green ratio. We observed increasing subG1 and S populations and decreasing G1 and G2/M populations with increasing amounts of glucosamine, while increasing amounts of silibinin led to increases in subG1, S, and G2/M populations and decreases in G1 populations in TC28a2 cells. MTT data showed that both glucosamine and silibinin induced cytotoxicity in TC28a2 cells in a dose-dependent manner. Regarding endoplasmic reticulum stress, both compounds induced the expression of CHOP and increased the level of p-eIF2α/eIF2α. With respect to O-GlcNAcylation status, glucosamine and silibinin both reduced the levels of O-GlcNAc transferase and hypoxia-inducible factor 1 alpha. Furthermore, we examined proteins and mRNAs related to these processes. In summary, our findings demonstrated that these compounds differentially modulated cellular proliferation, mitochondrial and cytosol ROS generation, the mitochondrial membrane potential, the cell cycle profile, and autophagy. Therefore, we conclude that glucosamine and silibinin not only mediate glycosylation modifications but also regulate cellular processes in human chondrocytes.

## 1. Introduction

The prevalence of osteoarthritis surpasses that of any other form of arthritis [1,2]. Rheumatoid arthritis affects only a fraction of the number of people afflicted with osteoarthritis. While joint symptoms are common to both, the underlying cause differs significantly. Osteoarthritis is a result of the gradual mechanical deterioration of joints, whereas rheumatoid arthritis is an autoimmune disease whereby the immune system attacks the body’s own joints. Glucosamine, an amino monosaccharide, has been used as a dietary supplement for over fifty years to alleviate discomfort caused by rheumatoid arthritis or osteoarthritis [3]. It is believed to be an anabolic agent, as it is a precursor of cartilage glycosaminoglycans. Osteoarthritis is a prevalent condition characterized by the loss of articular cartilage due to degenerative changes in the joint. Cartilage degradation is marked by an early phenomenon of the proteolytic loss of aggrecan, which forms large aggregates with hyaluronan in the extracellular matrix (ECM) [4]. Unlike ADAMTS4 (A Disintegrin and Metalloproteinase with Thrombospondin motifs 4), ADAMTS5 is N-glycosylated and does not bind to the cell surface but has been shown to reside in the ECM. Glucosamine has been found to impair furin’s function by modifying the post-translational glycosylation of furin, resulting in a decrease in its conversion to active furin and secondarily preventing the ADAMTS zymogen from being activated [5].

In vitro studies showed that glucosamine could effectively suppress the catabolic responses of cartilage explants and chondrocytes to retinoic acid and interleukin-1 (IL-1), which might induce the expression of chemokines, growth factors, inflammatory cytokines, and proteins involved in prostaglandin E2 and nitric oxide synthesis [6]. Additionally, glucosamine might block the IL-1-induced expression of matrix-specific proteases, including matrix metalloproteinase-3 (MMP-3), MMP-9, MMP-10, MMP-12, and ADAMTS1 [7]. Given that almost all receptors on the cell membrane are N-linked glycosylated proteins, N-glycosylation plays an important role in proteins’ stability and functions. The global inhibition of protein N-glycosylation induced by glucosamine is believed to be the basic mechanism underlying its multiple biochemical and cellular effects [8]. Therefore, the glucosamine-induced inhibition of gp130 N-glycosylation can reduce IL-6’s binding to cells, which suppresses the IL-6/Janus Kinase (JAK)/Signal Transducer and Activator of Transcription 3 (STAT3) signaling pathway. Furthermore, the effect of glucosamine on other N-glycosylated proteins might be one of the basic mechanisms underlying its anti-cancer activity.

Silibinin, extracted from milk thistle, is a powerful component of the silymarin complex that has been proven to provide numerous health benefits [9,10]. It shows excellent antioxidant effects and estrogenic activity, and it also modulates drug transporter (P-glycoprotein) function. Silibinin has been found to have specific effects on gene expression by suppressing the activity of the transcription factor NF-κB. Silibinin can inhibit proinflammatory cytokine-related signaling pathways, making it a potent anti-inflammatory agent. Moreover, it has been shown to have remarkable cardio-protective, neuroprotective, and hepato-protective activities [11]. Its ability to modify the degree of glycosylation of target proteins like Intracellular Adhesion Molecule-1 (ICAM-1) and PDGF receptor beta (PDGFRβ) makes it an essential component in the treatment of various diseases [12]. Silibinin can also inhibit global protein synthesis, making it a viable option for treating conditions associated with protein synthesis dysregulation [13].

The modification of transcription factors by O-linked N-acetylglucosamine (O-GlcNAc) plays a vital role in regulating gene expression in different tissues [14,15]. Through its target proteins, O-GlcNAc modulates a wide range of cellular functions including cell signaling, proteolytic degradation, and translation. This modification involves two enzymes; O-GlcNAc transferase (OGT) adds the GlcNAc, while O-GlcNAcase (OGA) removes it. The sensitivity of O-GlcNAcylation to nutrient levels in the cellular environment is due to the synthesis of UDP-GlcNAc through the hexosamine biosynthetic pathway (HBP). When cells are treated with glucosamine, which enhances the HBP flux, O-GlcNAcylation levels temporarily increase, resulting in lower OGT and higher OGA protein levels [16]. 

The avascular cartilage is integral in maintaining healthy joints and remains in a constant hypoxic state throughout life [17]. Damage to cartilage associated with osteoarthritis may result in deeper synovial fluid penetration, leading to an increase in oxygen supply. The chondrocyte, which is the resident cell type in articular cartilage, plays a unique role in ECM development, maintenance, and repair, and is highly specialized and metabolically active. Research on cartilage repair mechanisms related to chondrocyte biology and physiology has led to the development of the TC28a2 human chondrocyte cell line, which serves as a model cell line for studying the effects of different compounds on cartilage [18]. Glucosamine and silibinin are two widely used nutritional supplements that are known for their anti-inflammatory and antioxidant properties, which provide numerous health benefits. In our study, we aimed to investigate the functional roles of glucosamine and silibinin in cartilage homeostasis using the TC28a2 cell line. Our findings offer new insights into the benefits of glucosamine and silibinin, which are mediated not only through glycosylation modifications but also through cellular processes that promote healthy joints.

## 2. Results

### 2.1. The Effects of Glucosamine and Silibinin on the Membrane Proteins and Related Signaling Pathways Were Examined in Human TC28a2 Chondrocyte Cells

**Figure 1:**
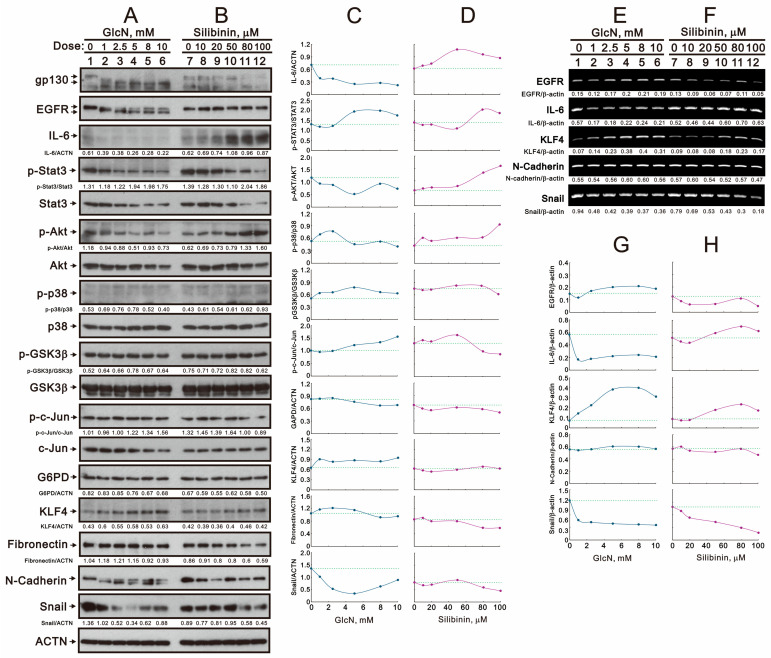
Effects of glucosamine and silibinin on the glycosylation of membrane proteins and signaling pathways in TC28a2 cells. TC28a2 cells were treated for 24 h with the indicated concentrations of (**A**,**E**) glucosamine and (**B**,**F**) silibinin, after which their lysates were subjected to (**A**,**B**) Western blot analysis using antibodies against the indicated proteins, with ACTN as the protein loading control; after which their lysates were subjected to (**E**,**F**) RT-PCR analysis using primer pairs for the indicated mRNAs, with *β-actin* mRNA as the mRNA loading and internal control. The protein and mRNA bands were quantified through pixel density scanning and evaluated using Image J, version 1.44a (http://imagej.nih.gov/ij/) (accessed on 1 February 2024). (**C**,**D**) We plotted the ratios of protein to ACTN and phosphated protein to total protein. (**G**,**H**) We plotted the ratios of mRNA/*β-actin*. The dashed line was based on the band density of the vehicle control.

We aimed to investigate the functional roles of glucosamine and silibinin in cartilage homeostasis using the TC28a2 cell line. N-glycosylation plays an important role in proteins’ stability and functions. The global inhibition of protein N-glycosylation is induced by glucosamine and silibinin. Hence, we treated TC28a2 cells with various concentrations of glucosamine and silibinin for 24 h and analyzed target proteins using Western blotting (Figure 1A–D). Our data showed that glucosamine consistently inhibited the N-glycosylation of gp130, epidermal growth factor receptor (EGFR), and N-cadherin and was further degraded in TC28a2 cells. Based on the gp130-STAT3-AKT pathway, we also observed that glucosamine led to a decrease in IL-6, p-STAT3, STAT3, p-AKT, AKT, c-Jun, glucose-6-phosphate dehydrogenase (G6PD), fibronectin, and Snail; an increase in Krüppel-like factor 4 (KLF4); and no effect on p38, p-p38, glycogen synthase kinase 3β (GSK3β), p-GSK3β, G6PD, or p-c-Jun (Figure 1A). Silibinin decreased the gp130, EGFR, p-STAT3, STAT3, AKT, p-c-Jun, fibronectin, N-cadherin, and Snail proteins; increased the IL-6, p-AKT, and KLF4 proteins; and had no effect on G6PD in TC28a2 cells (Figure 1B). The quantitative data indicated that the ratios of p-STAT3/ STAT3 and p-c-Jun/c-Jun increased due to the decrease in total STAT3 and c-Jun proteins induced by glucosamine (Figure 1C). The change trends of IL-6, p-AKt/AKt, KLF4, and Snail differed between glucosamine and silibinin (Figure 1C,D). Additionally, we further examined the down-shift (or lower molecular weight) N-glycosylation proteins, EGFR and N-cadherin in Figure 1A to determine whether they were mediated through the alternative splicing pathway to produce lower molecular weight isoform proteins. Our RT-PCR analysis demonstrated that *EGFR* mRNA was induced and *N-Cadherin* mRNA remained unchanged by glucosamine (Figure 1E,G). For the study of silibinin, the change trends of *EGFR* and *IL-6* mRNAs differed from their proteins (Figure 1F,H). We observed an increase in *KLF4* mRNA and a decrease in *Snail* mRNA when TC28a2 cells were treated with glucosamine and silibinin, and these trends were consistent with their proteins. Our *EGFR* and *N-cadherin* mRNA data also suggested that the subsequent degradation of N-glycosylated proteins by glucosamine was not mediated through transcriptional regulation. However, the degradation of EGFR and N-cadherin by silibinin was mediated through the transcriptional level.

### 2.2. The Effects of Glucosamine and Silibinin on Cellular Proliferation, Cytosolic and Mitochondrial ROS, Mitochondrial Membrane Potential, and Autophagy Were Examined in Human TC28a2 Chondrocyte Cells

**Figure 2:**
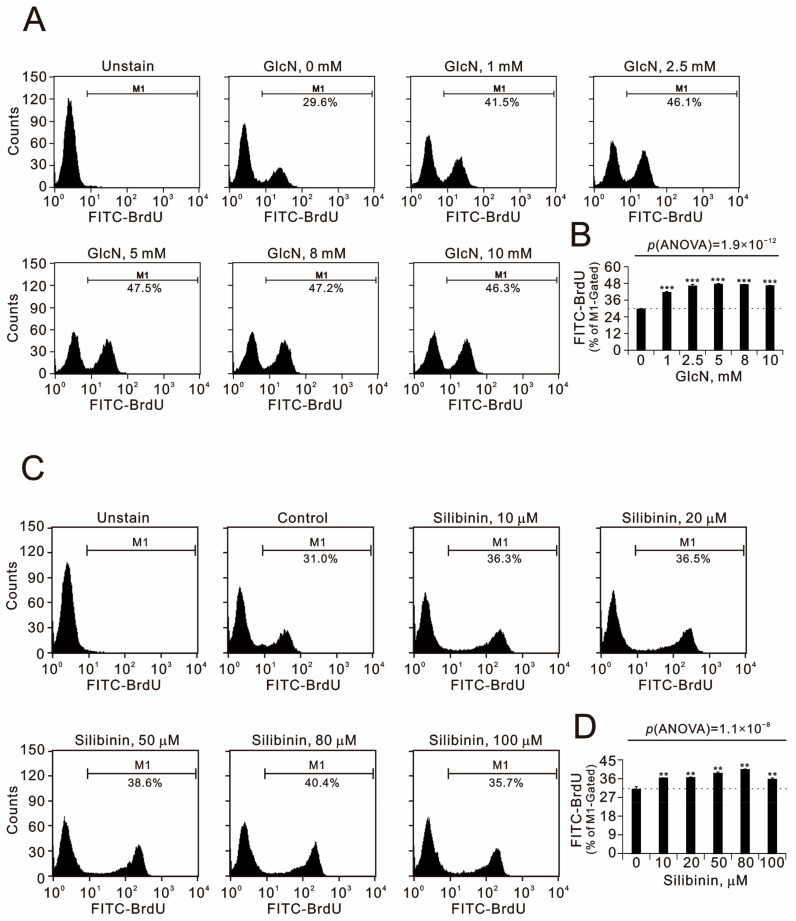
Effects of glucosamine and silibinin on proliferation of TC28a2 cells. TC28a2 cells were treated for 24 h with the indicated concentrations of (**A**,**B**) glucosamine and (**C**,**D**) silibinin. (**A**,**C**) After treatment, the cells were stained with BrdU, incorporated into newly synthesized DNA, progressed through the S phase (DNA synthesis), and then analyzed using flow cytometry with reference to the BrdU incorporation level. (**B**,**D**) The results are representative of three independent experiments. Bars depict the means ± SDs. ** *p* < 0.01 and *** *p* < 0.001 (Student’s *t*-tests).

**Figure 3:**
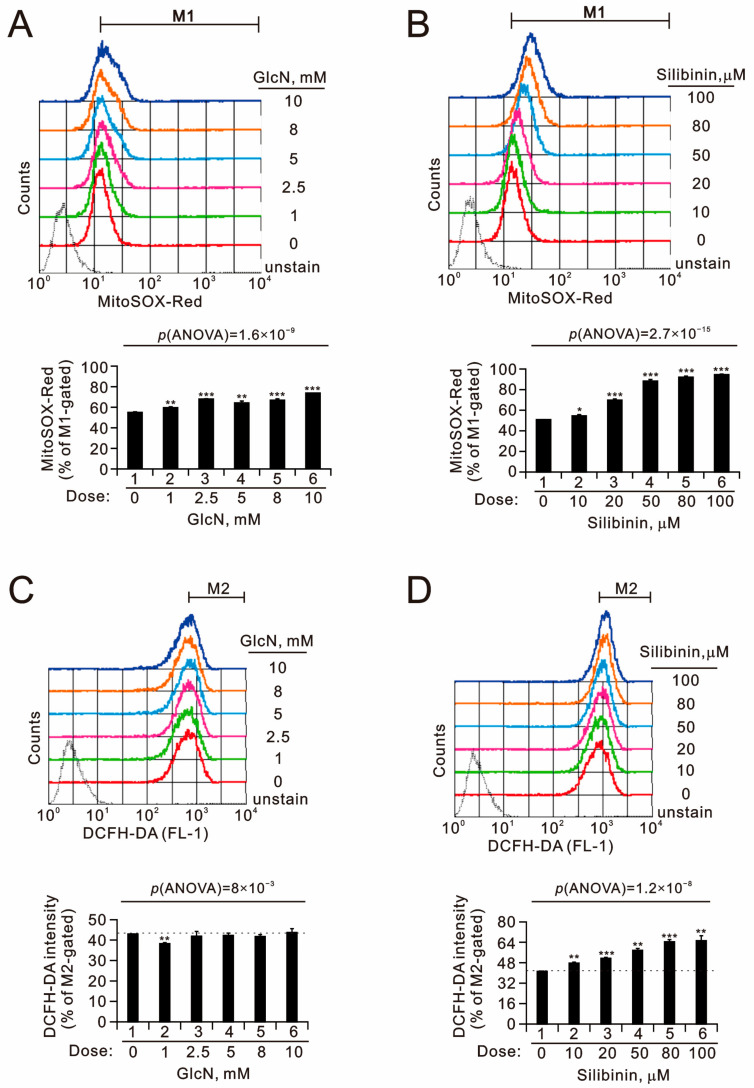
Effects of glucosamine and silibinin on ROS levels in TC28a2 cells. (**A**–**D**) TC28a2 cells were treated with the indicated concentrations of (**A**,**C**) glucosamine and (**B**,**D**) silibinin for 24 h. (**A**,**B**) Mitochondrial ROS and (**C**,**D**) intracellular ROS levels were assessed using DCFH-DA and MitoSOX Red staining with flow cytometry. Bars depict the means ± SDs of three independent experiments. * *p* < 0.05; ** *p* < 0.01; *** *p* < 0.001 (Student’s *t*-tests).

Glucosamine is believed to be an anabolic agent, as it is a precursor of cartilage glycosaminoglycans. We sought to examine the effects of glucosamine and silibinin on the cellular proliferation of chondrocytes. Our BrdU cellular proliferation data showed that glucosamine and silibinin both significantly induced the proliferation of TC28a2 cells in a dose-dependent manner (Figure 2). The maximal effective concentration of glucosamine for the proliferation of TC28a2 cells was 2.5 µM, and that of silibinin was 80 µM. The popular clinical application of glucosamine and silibinin is related to their antioxidative properties. Cellular reactive oxygen species (ROS) are primarily produced in mitochondria and partly in the cytoplasm. We applied MitoSOX and DCFH-DA dyes for the detection of mitochondrial and cytosol ROS, respectively, using flow cytometry analysis (Figure 3). Our data showed that glucosamine suppressed the mitochondrial ROS generation at 1 µM but had no effect at higher concentrations. However, glucosamine induced cytosolic ROS generation at all the tested concentrations. The tested silibinin concentrations all significantly induced mitochondrial and cytosol ROS generation in TC28a2 cells.

**Figure 4:**
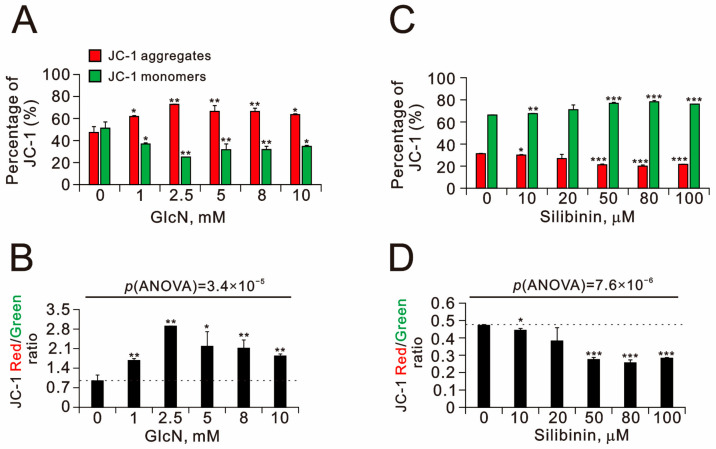
Effects of glucosamine and silibinin on the mitochondrial membrane potential of TC28a2 cells. (**A**,**B**) TC28a2 cells were treated with the indicated concentrations of (**A**,**B**) glucosamine and (**C**,**D**) silibinin for 24 h. Mitochondrial membrane potential was assessed using JC-1 staining with flow cytometry. (**A**,**C**) The percentages of red and green fluorescence intensities are plotted. (**B**,**D**) The red/green fluorescence intensity ratios were measured and are plotted. Bars depict the means ± SDs of three independent experiments. * *p* < 0.05; ** *p* < 0.01; *** *p* < 0.001 (Student’s *t*-tests).

The ROS induced by damaged mitochondria may lead to the induction of mitophagy and elimination of damaged organelles to reduce ROS levels. The loss of mitochondrial membrane potential was initially considered to be a clue to mitophagy [19]. We then examined the effects of glucosamine and silibinin on mitochondrial membrane potential in TC28a2 cells using JC-1 and flow cytometry (Figure 4). We utilized the JC-1 dimer form in mitochondria with red fluorescence and JC-1 monomer in the cytoplasm with green fluorescence to measure mitochondrial depolarization as indicated by a decrease in the red/green fluorescence intensity ratio. Our data showed that glucosamine significantly increased the red JC-1 aggregates, resulting in a dose-dependent increase in the red/green fluorescence intensity ratio. In TC28a2 cells, all the tested silibinin concentrations significantly increased the green monomers, resulting in decreases in the red/green ratio.

**Figure 5:**
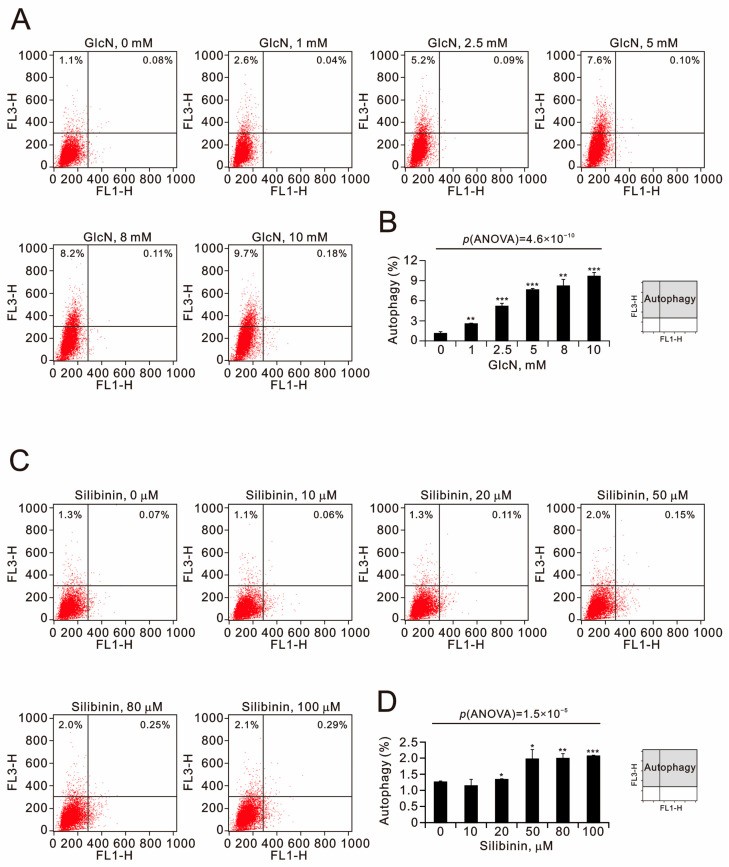
Effects of glucosamine and silibinin on autophagy in TC28a2 cells. (**A**,**B**) TC28a2 cells were treated with the indicated concentrations of (**A**,**B**) glucosamine and (**C**,**D**) silibinin for 24 h. Acridine orange (1 µg/mL) staining was used to identify autophagic cells via FACS. (**A**,**C**) Acidic vesicular organelles were detected and quantified using acridine orange staining and measured using flow cytometry. (**B**,**D**) The intensity of the red fluorescence (y-axis, FL3-H) was proportional to the degree of acidity and the volume of acidic vesicular organelles, including autophagic vacuoles. The values refer to the percentages of cells with a significant proportion of acidic vesicular organelles. The results are representative of three independent experiments. Bars depict the means ± SDs. * *p* < 0.05; ** *p* < 0.01; *** *p* < 0.001 (Student’s *t*-tests).

The initiation of autophagy is regulated by the level of ROS [20]. Acridine orange (AO) has been trapped at high concentrations in acidic vesicles with a red fluorescence emission [21]. Here, we quantified acidic vesicular organelles using flow cytometry to measure the effects of glucosamine and silibinin on the formation of autophagolysosomes in TC28a2 cells (Figure 5). Our data revealed that glucosamine significantly increased the percentage of acidic vesicular organelles from 1.1% to 9.7%, and silibinin only increased the percentage of acidic vesicular organelles from 1.3% to 2.3%.

### 2.3. The Effects of Glucosamine and Silibinin on the Cell Cycle Profile and Cytotoxicity Were Examined in Human TC28a2 Chondrocyte Cells

**Figure 6:**
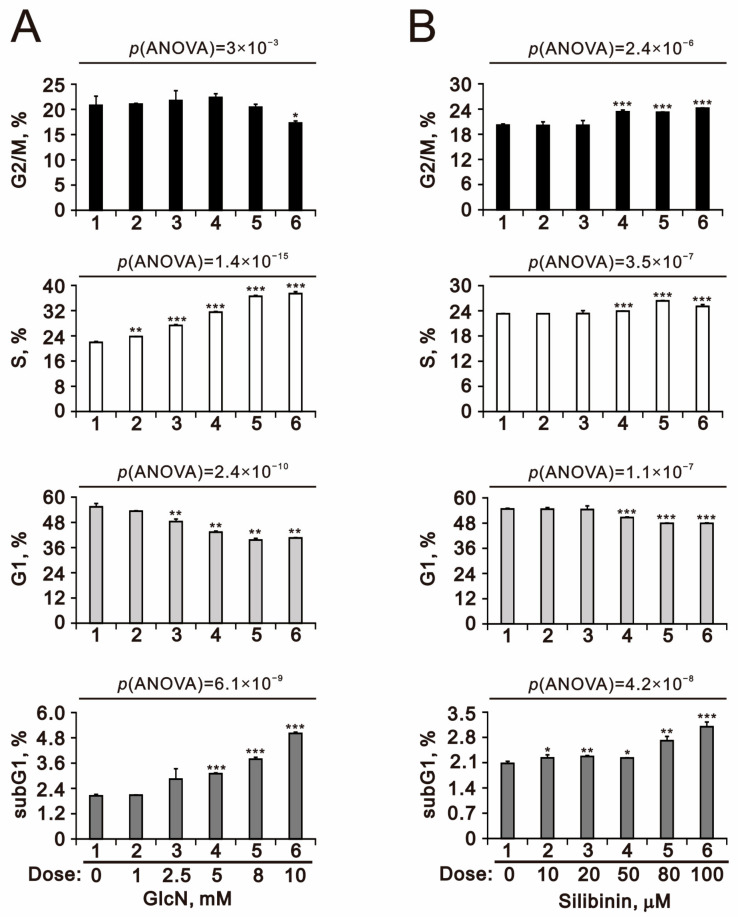
Effects of glucosamine and silibinin on the cell cycle of TC28a2 cells. (**A**,**B**) TC28a2 cells were treated with the indicated concentrations of (**A**) glucosamine and (**B**) silibinin for 24 h. Cells were stained with propidium iodide (PI) and analyzed using flow cytometry. Bars depict the means ± SDs of three independent experiments. * *p* < 0.05; ** *p* < 0.01; *** *p* < 0.001 (Student’s *t*-tests).

**Figure 7:**
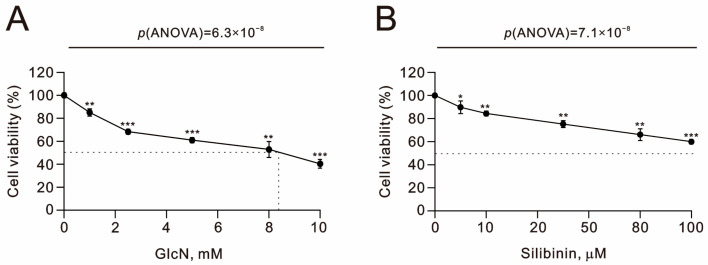
Effects of glucosamine and silibinin on cell viability in TC28a2 cells. (**A**,**B**) TC28a2 cells that were treated with the indicated concentrations of (**A**) glucosamine and (**B**) silibinin for 24 h. Control cells were cultured under identical conditions. Cell viability (metabolic activity) was measured using an MTT colorimetric assay. Data are presented as a percentage of the control. Bars depict the means ± SDs. * *p* < 0.05; ** *p* < 0.01; *** *p* < 0.001 (Student’s *t*-tests).

Our current data demonstrated that glucosamine and silibinin had differential effects on ROS, the mitochondrial membrane potential, and autophagy. Next, we examined the effects of both on the cell cycle profile and cytotoxicity in TC28a2 cells. We applied podium iodine (PI) to examine the effects of glucosamine and silibinin on the cell cycle profile of TC28a2 cells using flow cytometry (Figure 6). We observed increasing subG1 and S populations and decreasing G1 and G2/M populations with increasing amounts of glucosamine. Increasing amounts of silibinin led to increases in subG1, S, and G2/M populations and decreases in G1 populations in TC28a2 cells. We further applied MTT survival analysis to examine the cytotoxic effects of glucosamine and silibinin on TC28a2 cells. Our MTT (thiazolyl blue tetrazolium bromide) data showed that glucosamine and silibinin significantly induced cytotoxicity in TC28a2 cells in a dose-dependent manner. The half-lives of glucosamine and silibinin were 8.4 mM and over 100 μM, respectively (Figure 7).

### 2.4. The Effects of Glucosamine and Silibinin on Specific Proteins and mRNAs Related to Cellular Stresses Were Examined in Human TC28a2 Chondrocyte Cells

**Figure 8:**
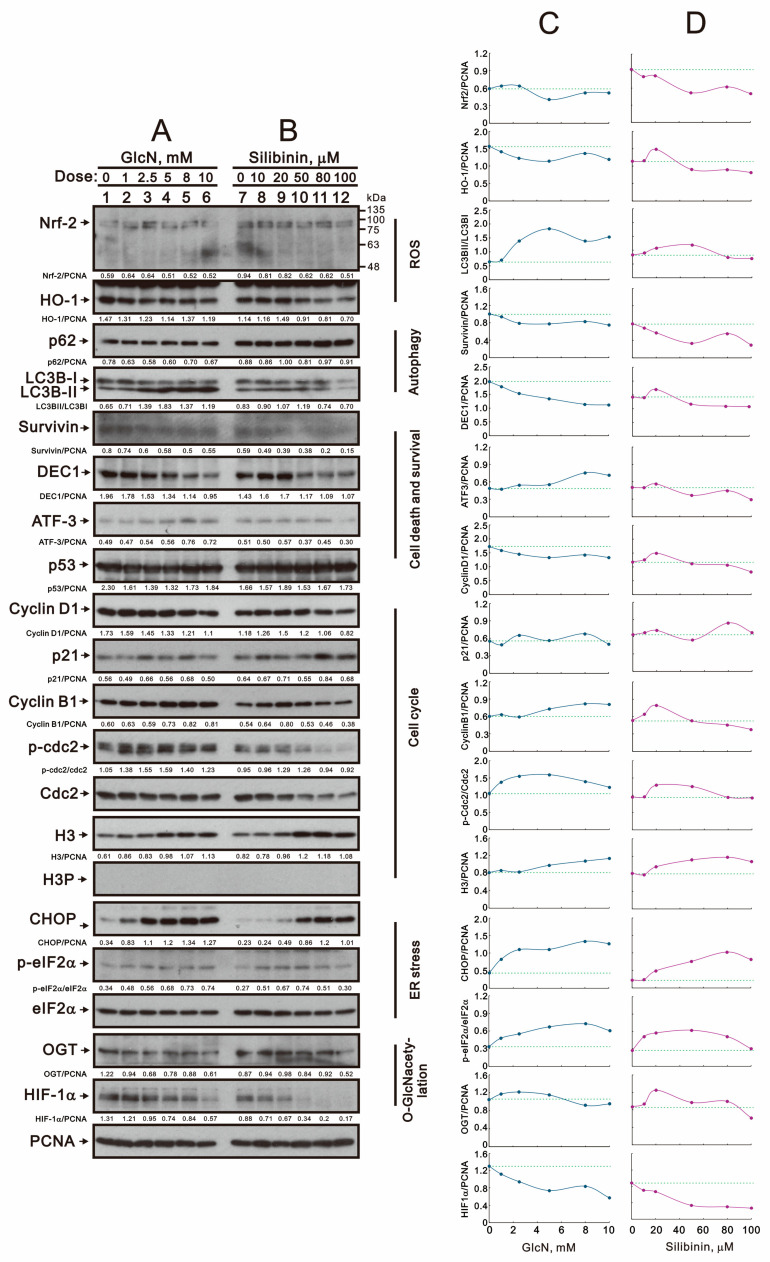
Effects of glucosamine and silibinin on the proteins related to various cellular stresses in TC28a2 cells. (**A**,**B**) TC28a2 cells were treated for 24 h with the indicated concentrations of (**A**) glucosamine and (**B**) silibinin, after which their cell lysates were subjected to Western blot analysis using antibodies against the indicated proteins, with PCNA as the protein loading control. (**C**,**D**) The protein bands were quantified through pixel density scanning and evaluated using Image J, version 1.44a (http://imagej.nih.gov/ij/) (accessed on 1 February 2024). We plotted the ratios of protein to PCNA and phosphated protein to total protein. The dashed line was based on the band density of the vehicle control.

Our current data demonstrated that glucosamine and silibinin had differential effects on ROS, the mitochondrial membrane potential, autophagy, the cell cycle profile, and cytotoxicity. We further examined related proteins with these processes using Western blotting in TC28a2 cells (Figure 8A,B). Regarding ROS status, glucosamine and silibinin decreased the expression of nuclear factor erythroid 2-related factor 2 (Nrf-2) and its target heme oxidase 1 (HO-1) proteins. In terms of autophagy status, glucosamine increased the level of light chain 3B (LC3B) II and the LC3B II/LC3B I ratio; however, silibinin decreased the levels of LC3B II and LC3B I and increased the level of p62 proteins. Regarding cell death and survival status, glucosamine decreased the levels of survivin and differentiated embryo-chondrocyte expressed gene 1 (DEC1) and increased the level of activating transcription factor 3 (ATF3); silibinin decreased the levels of survivin, DEC1, and ATF3. Both had no effect on the level of the p53 protein. Regarding cell cycle status, glucosamine decreased the levels of cyclin D1 and p21 and increased the levels of cyclin B1, p-cdc2, and histone H3; silibinin decreased the levels of cyclin D1, p-cdc2, and cdc2 and increased the levels of p21, cyclin B1, and histone H3. Both had no effect on phosphorylated H3 at serine 10 (H3P). Regarding endoplasmic reticulum (ER) stress, glucosamine and silibinin both induced the expression of C/EBP homologous protein (CHOP) and increased the level of p-eIF2α/eIF2α. Regarding O-GlcNAcylation status, glucosamine and silibinin both reduced the levels of OGT and hypoxia-inducible factor 1 alpha (HIF-1α). We furthre quantified, normalized, and plotted these Western blotting data (Figure 8C,D). In general, our quantified data demonstrated that glucosamine and silibinin both decreased the amounts of Nrf2, HO-1, DEC1, cyclin D1, and HIF-1α and increased the amounts of LC3BII/LC3BI, p21, cyclin B1, p-Cdc2/Cdc2, H3, CHOP, p-eIF2α/eIF2α, and OGT. The only difference was ATF3 induced by glucosamine and suppressed by silibinin.

**Figure 9:**
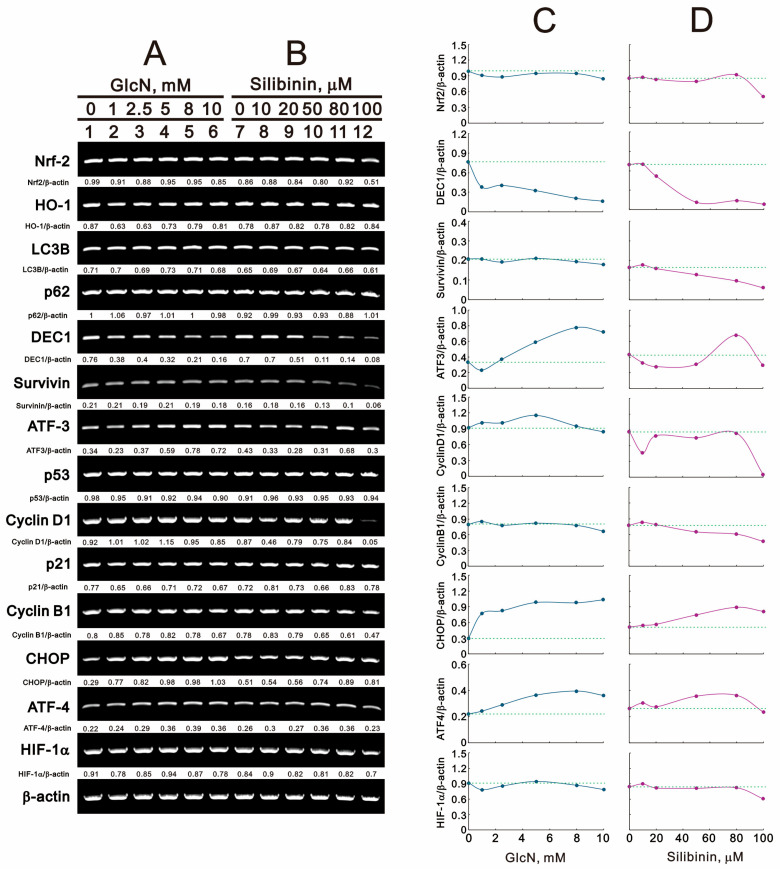
Effects of glucosamine and silibinin on cellular stress mRNAs in TC28a2 cells. (**A**,**B**) TC28a2 cells were treated for 24 h with the indicated concentrations of (**A**) glucosamine and (**B**) silibinin, after which their lysates were subjected to RT-PCR analysis using primer pairs for the indicated mRNAs, with *β-actin* mRNA as the mRNA loading and internal control. The mRNA bands were quantified through pixel density scanning and evaluated using Image J, version 1.44a (http://imagej.nih.gov/ij/) (accessed on 1 February 2024). (**C**,**D**) The ratios of mRNA/*β-actin* were plotted. The dash line was plotted based on the band density of vehicle.

We further examined the levels of target mRNAs using RT-PCR analysis (Figure 9A,B). Glucosamine and silibinin both induced the expression of *CHOP* mRNA and suppressed the expression of *DEC1* mRNA in TC28a2 cells. Glucosamine selectively induced *ATF3* mRNA expression, and silibinin suppressed the expression of *Nrf2*, *cyclin D1*, and *cyclin B1* mRNAs. Glucosamine and silibinin both had no effect on the expression of *HO-1*, *LC3B*, *p62*, *p53*, *p21*, *ATF-4*, and *HIF-1α* mRNAs. We proceeded to quantify, normalize, and graph the RT-PCR data (Figure 9C,D). Our analysis revealed that both glucosamine and silibinin decreased the levels of *Nrf2*, *DEC1*, *Survivin*, *cyclin B1*, and *HIF-1α*, while increasing the levels of *CHOP* and *ATF4*. Additionally, glucosamine induced *ATF3* and *cyclin D1*, whereas silibinin suppressed their expression.

## 3. Discussion

The articular cartilage responds to various physiological stresses, including mechanical stress, to maintain homeostasis at the molecular level. However, if this balance is disrupted, osteoarthritis develops and progresses. Protein glycosylation can be classified into three types: N-linked glycosylation, O-linked glycosylation, and glycosylphosphatidyl inositol anchored linkage [22]. It is interesting to note that the reduction in the proteasome activator PA28γ brought about by the glucosamine-induced O-GlcNAc modification leads to the suppression of proteasomal activity, which leads to cell death in ALVA41 cells [23]. Glucosamine’s global inhibition of protein N-glycosylation may be the fundamental mechanism underlying its various biochemical and cellular effects [24]. Similarly, silibinin modulates the glycosylation of several target proteins to exert its effects. In this study, we observed that glucosamine suppressed the N-glycosylation of the gp130, EGFR, and N-cadherin proteins. Furthermore, glucosamine and silibinin differentially decreased and increased target proteins such as gp130, Snail, and KLF4 in TC28a2 cells. Our findings also demonstrated that these compounds differentially modulated cellular processes such as proliferation, mitochondrial and cytosol ROS generation, the mitochondrial membrane potential, the cell cycle profile, and autophagy. We also examined proteins and mRNAs related to these processes, and we conclude that glucosamine and silibinin mediate not only glycosylation modifications but also cellular processes in human chondrocytes.

O-GlcNAcylation increases α-ketoglutarate, HIF-1α hydroxylation, and HIF-1αs’ interaction with the von Hippel–Lindau protein, which leads to HIF-1α’s degradation. Hence, O-GlcNAc modification controls glycolysis in cancer cells and induces ER stress by modifying HIF-1α and its transcriptional target glucose transporter 1 (GLUT1) [25]. High glucose-stimulated HIF-1α-GLUT1–advanced glycation end-product signaling in rat fibroblast-like synoviocytes subsequently activated ER stress and the release of proinflammatory factors from the synovium and finally induced inflammation and the degeneration of articular cartilage [26]. In osteoarthritis, capsiate treatment is related to decreased HIF-1α expression and oxidative stress levels [27]. miR-411 promotes chondrocyte autophagy by targeting HIF-1α [28]. Silibinin inhibits cellular glucose uptake by directly interacting with GLUT transporters [29]. Our current data show that both glucosamine and silibinin reduced the levels of the HIF-1α protein, not mRNA, accompanied by the induction of ER stress and differential levels of autophagy and ROS in TC28a2 cells. Additionally, it has been demonstrated that glucosamine and silibinin do not affect the HIF-1α protein degradation rate or *HIF-1α* steady-state mRNA level but do suppress HIF-1α expression at the translational level. One study showed that the expression of OGT and elevated levels of HIF-1α are correlated with poor patient outcomes in breast cancers [25]. Future research will aim to verify these regulatory mechanisms of HIF-1α in human chondrocytes. In contrast, local treatment with a selective hypoxia mimetic in the joint restores the disruptor of telomeric silencing 1-like (DOT1L) function and could be an attractive therapeutic strategy for osteoarthritis [30]. DOT1L, which methylates lysine 79 of histone H3, is one of the targets of HIF-1α and contributes to the protective effects of hypoxia on osteoarthritis. It would be interesting to investigate the effects of glucosamine and silibinin on HIF-1α suppression and the status of epigenetics in TC28a2 cells.

N-linked glycosylation plays an important role in the proper folding and trafficking of many proteins in the ER. HBP activation promotes ER stress, lipid accumulation, and inflammation in cells grown in high-glucose media [31]. Hyperglycemia or exogenous glucosamine supplementation increases flux through the HBP and is associated with the induction of ER stress and unfolded protein response activation in vascular cells as well as the development of hepatic steatosis and accelerated atherosclerosis in apoE^−/−^ mice [31,32,33]. The laboratory of Dr. Delbrel provides evidence that a hypoxic microenvironment and the stabilization of HIF-1α induce ER stress in alveolar epithelial cells; the expression of CHOP, a pro-apoptotic factor currently used as a marker of ER stress in idiopathic pulmonary fibrosis; and subsequent apoptosis [34]. In our study, we found a decrease in the HIF-1α protein, not its mRNA, accompanied by the induction of ER stress and apoptosis, as evidenced by increased populations in the subG1 phase and decreased DEC-1 protein. However, the amounts of CHOP protein and mRNA were increased by glucosamine and silibinin, suggesting that the HIF-1α protein plays a repressive role in TC28a2 cells. Glucosamine and silibinin apparently disrupted N-linked glycosylation, leading to an increase in misfolded proteins in the ER, inducing ER stress in our experimental conditions. In addition, increased O-GlcNAc modification is related to the attenuation of the unfolded protein response and ER stress-induced cardiomyocyte death [35]. Snail is stabilized by O-GlcNAc modification in hyperglycemic conditions [36]. Our data showed that glucosamine and silibinin both suppressed the expression of the Snail protein and mRNA with the induction of ER stress, suggesting that O-GlcNAc modification might be also disrupted as well as N-link glycosylation in TC28a2 cells. The avascular cartilage is integral in maintaining healthy joints and remains in a constant hypoxic state throughout life [17]. The hypoxia and ER stress in our working system remain to be investigated because of the well-known model of the induction of ER stress via ATF4 by hypoxia [37,38]. In TC28a2 cells, how glucosamine and silibinin change the functional role of the HIF-1α protein from activator to repressor and whether ATF4 is involved in the regulation of CHOP expression or not can be the subject of future research. This might help to explain the distinct functional roles of the HIF-1α and ATF4 proteins in distinct cell types.

Autophagy dysfunction results in the impaired degradation of organelles and proteins damaged by oxidative stress, leading to the induction of cell death [39]. Aging is a major risk factor for osteoarthritis, and chondrocytes in aged individuals exhibit senescent phenotypes, such as reduced resistance to oxidative stress and impaired cellular homeostasis due to autophagy dysfunction [40,41,42]. Enhancing autophagy might protect against aging-related cell dysfunction and reduce the risk of osteoarthritis. Our findings suggest that glucosamine and silibinin enhance autophagy and ROS generation, leading to the induction of cell death in TC28a2 cells. We will examine whether these effects can be replicated in aged TC28a2 cells and whether they improve autophagy, ROS generation, and the induction of cell death. During osteoarthritis, chondrocytes exhibit increased levels of various senescence markers, such as senescence-associated β-galactosidase activity and the accumulation of p16 [40]. We will investigate whether glucosamine and silibinin have any effect on these markers in aged TC28a2 cells. We believe that further details on how glucosamine and silibinin affect healthy, osteoarthritis, and aged chondrocytes will provide the final answer.

Based on our current findings, glucosamine and silibinin appear to have different targets for post-translational modifications, and the proteins they modify are not specific to chondrocyte cells. This suggests that the functions in chondrocytes may or may not be directly mediated through the glycosylation of their target proteins. Furthermore, in addition to modulating autophagy and ROS, glucosamine and silibinin induced cellular proliferation. The suppression of the G1 phase and the prolongation of the S phase are correlated with the increased cell proliferation, which subsequently leads to the synthesis of more unfolded proteins and the induction of ER stress by glucosamine and silibinin in our current study. ER stress can also result from the inhibition of post-translational modifications by glucosamine and silibinin. Therefore, the health benefits of glucosamine and silibinin as nutritional supplements should be carefully evaluated, considering the cascade of well-known targets and the unidentified targets with post-translational modifications.

## 4. Materials and Methods

### 4.1. Cell Culture and Reagents

The TC28a2 human chondrocyte cell line was purchased from Merck (Elkton, VA, USA). The TC28a2 cell line was established by transfecting primary cultures (day 5) of costal cartilage from a 15-year-old female with a retroviral vector expressing simian virus SV40 large T antigen. TC28a2 cells were cultured in DMEM/F-12 (Corning, Corning City, NY, USA), containing 10% fetal bovine serum (FBS) and 1% penicillin–streptomycin (Thermo Fisher Scientific, Carlsbad, CA, USA). Acridine orange, 2′,7-dichlorofluorescein diacetate (DCFH-DA), glucosamine, and silibinin were purchased from Sigma-Aldrich (St. Louis, MO, USA)

### 4.2. Western Blotting Analysis

Cells were lysed with lysis buffer (100 mM tris–HCl (pH 8.0), 150 mM NaCl, 0.1% SDS, and 1% Triton 100) at 4 °C. The protein concentrations in the lysates were measured using Bio-Rad Protein Assay Dye Reagent Concentrate (Bio-Rad Laboratories, Hercules, CA, USA). The protein lysates were prepared with 4× protein loading dye and denatured at 95 °C for 10 min, separated on 12% SDS-PAGE gels, and blotted onto PVDF membranes (Immobilon-P; Millipore, Bedford, MA, USA) using a Bio-Rad Semi-Dry Transfer Cell. The blots were then incubated with primary antibodies against α-actinin (ACTN, H-2, sc-17829, 1:5000 dilution), p62 (D-3, sc-28359, 1:1000 dilution), Nrf-2 (H-6, sc-518033, 1:1000 dilution), p53 (DO-1, sc-126, 1:5000 dilution), Cyclin B1 (GNS1, sc-245, 1:1000 dilution), OGT (F-12, sc-74546, 1:1000 dilution), and PCNA (F-2, sc-25280, 1:5000 dilution) (Santa Cruz Biotechnology, Santa Cruz, CA, USA); LC3B (#2775, 1:1000 dilution), EGFR (#4267, 1:1000 dilution), IL6 (#12153, 1:1000 dilution), p-Stat3 (#9131, 1:1000 dilution), Stat3 (#9139, 1:1000 dilution), p-Akt (#9271, 1:1000 dilution), Akt (#4691, 1:1000 dilution), p-p38 (#9211, 1:1000 dilution), p38 (#9212, 1:1000 dilution), p-GSK3β (#5558, 1:5000 dilution), GSK3β (#12456, 1:2000 dilution), p-c-Jun (#2361, 1:1000 dilution), c-Jun (#9165, 1:1000 dilution), G6PD (#12263, 1:1000 dilution), N-Cadherin (#13116, 1:1000 dilution), Snail (#3895, 1:1000 dilution), ATF-3 (#33593, 1:1000 dilution), p-cdc2 (#9111, 1:1000 dilution), cdc2 (#9116, 1:1000 dilution), H3 (#9715, 1:2000 dilution), H3P (#9701, 1:1000 dilution), CHOP (#2895, 1:1000 dilution), p-eIF2α (#9721, 1:1000 dilution), eIF2α (#9722, 1:1000 dilution), and HIF1α (#14179, 1:1000 dilution) (Cell Signaling, Danvers, MA, USA); KLF4 (ab215036, 1:1000 dilution), Fibronectin (ab32419, 1:1000 dilution), Survivin (ab76424, 1:1000 dilution), cyclin D1 (ab134175, 1:1000 dilution), and p21 (ab109520, 1:1000 dilution) (Abcam, Cambridge, UK); HO-1 (ADI-SPA-895F, 1:1000 dilution) (Enzo Life Sciences Inc., Farmingdale, NY, USA); and DEC1 (A300-649A, 1:1000 dilution) (Bethyl Laboratories Inc., Montgomery, TX, USA). Thereafter, the blots were incubated with HRP-conjugated secondary antibodies (anti-mouse IgG (AP192P) and anti-rabbit IgG (AP132P), Merck-Millipore, Burlington, MA, USA). The immunoreactive proteins were detected using ECL™ Western Blotting Detection Reagent and Amersham Hyperfilm™ ECL (GE Healthcare, Chicago, IL, USA). The procedural details are described in our previous publications [43,44].

### 4.3. Fluorescence-Activated Cell Sorting (FACS) for Flow Cytometry Analyses of Cell Cycle Profiles, Proliferation, ROS, and Mitochondrial Membrane Potential

The cell proliferation was assessed using immunofluorescent staining with incorporated bromodeoxyuridine (BrdU) (BD Pharmingen™ BrdU Flow Kit, BD Biosciences, San Jose, CA, USA) and flow cytometry, according to the manufacturer’s instructions. Briefly, the cells were seeded in 6-well culture plates and treated with the selected drugs for 24 h. After incubation, the cells were stained with BrdU, harvested, washed with PBS, and then fixed and permeabilized before being stained using BrdU with fluorescent antibodies. The cells were resuspended in staining buffer, and an FITC-BrdU fluorescence analysis was performed using a FACSCalibur flow cytometer and Cell Quest Pro software (BD Biosciences).

Mitochondrial membrane potential was monitored using the MitoScreen (JC-1) kit (BD Biosciences). After being treated with tramadol, dead and live cells were collected, and JC-1 solution was added prior to the 15 min incubation. The cells were then washed twice with a binding buffer. Each sample was evaluated using the FACSCalibur flow cytometer and Cell Quest Pro software.

The intracellular and mitochondrial ROS levels were assessed using DCFH-DA and MitoSOX Red staining, respectively. Briefly, the treated cells were washed with PBS twice and incubated with 10 μM DCFH-DA or 5 μM MitoSOX Red (Thermo Fisher Scientific, M36008) at 37 °C for 30 min in the dark. Afterwards, the cells were washed once with PBS, and then the DCFH-DA or MitoSOX Red fluorescence intensity was analyzed on the FL-1 or FL-3 channel of the FACSCalibur flow cytometer using Cell Quest Pro software (BD Biosciences). The median fluorescence intensity of the vehicle was used as the starting point for M1 gating.

The cell cycle profiles were assessed by measuring the cellular DNA content using propidium iodide (PI) staining. After being treated with the drugs, the cells were trypsinized and washed with PBS. We resuspended the cell pellet in 1 mL of PBS, fixed the cells in 5 mL of 70% ice-cold ethanol, and then stored them at −30 °C overnight. The following day, the cells were washed twice with ice-cold PBS containing 1% FBS and centrifuged at 4 °C at 1000 rpm for 5 min. Then, they were stained with a PI staining solution (5 μg/mL PI in PBS, 0.5% Triton X-100, and 0.5 μg/mL RNase A) for 30 min at 37 °C in the dark. Each sample was analyzed using the FACSCalibur flow cytometer and Cell Quest Pro software (BD Biosciences). 

### 4.4. Flow Cytometric Quantification of Acidic Vesicular Organelles

The acidic compartments of the cells were detected using acridine orange (Sigma-Aldrich) staining and measured with flow cytometry. As the protonated form of acridine orange accumulates inside acidic vesicles, it is a marker for the final steps of the autophagy process. Briefly, the cells were treated with the indicated glucosamine or silibinin dosages for 24 h, stained with acridine orange (1 μg/mL) for 20 min at 37 °C, and then trypsinized for harvesting. Afterwards, the cells were washed once with PBS, resuspended in 400 μL of PBS, and then analyzed via flow cytometry (FACSCalibur, BD Biosciences). The excitation wavelength was 488 nm, and the fluorescence at 510–530 nm (green fluorescence, FL1) and 650 nm (red fluorescence, FL3) was detected. The data were analyzed using the CellQuest™ software program. The percentage of autophagic cells was calculated based on the number of cells present in the upper-left and upper-right quadrants.

### 4.5. Metabolic Activity Analysis

Cells were plated into 24-well culture plates and incubated for 1 day, after which fresh DMEM/F-12 medium containing glucosamine or silibinin was added to each well. The procedural details have been described in our previous publications [43,44]. The cells were incubated with the selected treatments for the indicated periods. Then, the cells were incubated in MTT solution (0.5 mg/mL) for 1 h at 37 °C. At the end of the reaction, DMSO was added to dissolve the formazan crystals that formed during the reaction. The absorbances at 570 nm and 650 nm were measured using a multimode microplate reader (Varioskan™ LUX, Thermo Fisher Scientific). The metabolic activity was calculated based on the absorbance ratio between the cells cultured with the selected drugs and the untreated controls, which were assigned a value of 100.

### 4.6. Reverse Transcription–Polymerase Chain Reaction (RT-PCR)

Total RNAs were isolated from TC28a2 cells using TRIzol reagent (Thermo Fisher Scientific). Reverse transcription for first strand cDNA synthesis was carried out using MMLV reverse transcriptase (Epicentre Biotechnologies, Madison, WI, USA) with 1 μg of total RNA for 60 min at 37 °C. The PCRs were run on a Veriti Thermal Cycler (Applied Biosystems, Waltham, MA, USA). The PCR primers are listed in Table 1 below:

### 4.7. Statistical Analyses

The values are expressed as the means ± SDs of at least three independent experiments. All the comparisons between groups were conducted using Student’s *t*-tests, and the comparison among multiple groups was conducted using analysis of variance (ANOVA) with SPSS 20.0 for Windows (SPSS, Chicago, IL, USA). The statistical significance was set to *p <* 0.05.

## 5. Conclusions

In our study, we aimed to investigate the functional roles of glucosamine and silibinin in cartilage homeostasis using the TC28a2 cell line. Glucosamine suppressed the N-glycosylation of the gp130, EGFR, and N-cadherin proteins. Furthermore, glucosamine and silibinin differentially decreased and increased target proteins such as gp130, Snail, and KLF4 in TC28a2 cells. Our findings also demonstrated that these compounds differentially modulated cellular processes such as proliferation, mitochondrial and cytosol ROS generation, the mitochondrial membrane potential, the cell cycle profile, and autophagy. We conclude that glucosamine and silibinin mediate through not only glycosylation modifications but also cellular processes in human chondrocytes.

## Figures and Tables

**Table 1:** The PCR primers.

Primer Name	Sequence (5′ → 3′)
*β-actin*	Forward: 5′-GTGGGGCGCCCCAGGCACCA-3′ Reverse: 5′-CTCCTTAATGTCACGCACGATTTC-3′
*ATF3*	Forward: 5′-GAGGATTTTGCTAACCTGAC-3′ Reverse: 5′-TAGCTCTGCAATGTTCCTTC-3′
*N-Cadherin*	Forward: 5′-CCATTAGCCAAGGGAATTCAGC-3′ Reverse: 5′-GGTCTGGAGTTTCGCAAGTCTC-3′
*CHOP*	Forward: 5′-CATTGCCTTTCTCCTTCGGG-3′ Reverse: 5′-GCCGTTCATTCTCTTCAGCT-3′
*Cyclin B1*	Forward: 5′-GTTGATACTGCCTCTCCAAG-3′ Reverse: 5′-CTTAGTATAAGTGTTGTCAGTCAC-3′
*cyclin D1*	Forward: 5′-ATGGAACACCAGCTCCTGTGCTGC-3′ Reverse: 5′-TCAGATGTCCACGTCCCGCACGTCGG-3′
*DEC1*	Forward: 5′-GTACCCTGCCCACATGTACC-3′ Reverse: 5′-GCTTGGCCAGATACTGAAGC-3′
*EGFR*	Forward: 5′-GCTTTGGTGCCACCTGCGTG-3′ Reverse: 5′-CTCCATCACTTATCTCCTTGAG-3′
*HIF-1a*	Forward: 5′-GAACCTGATGCTTTAACT-3′ Reverse: 5′-CAACTGATCGAAGGAACG-3′
*HO-1*	Forward: 5′-ATGCCCCAGGATTTGTCAGAG-3′ Reverse: 5′-AGGGCTTTCTGGGCAATCTTT-3′
*IL-6*	Forward: 5′-ATGAACTCCTTCTCCACAAGCGC-3′ Reverse: 5′-CTACATTTGCCGAAGAGCCCTCA-3′
*KLF4*	Forward: 5′-CTTGAGGAAGTGCTGAGCAG-3′ Reverse: 5′-CGGTAGTGCCTGGTCAGTTC-3′
*LC3B*	Forward: 5′-AGCAGCATCCAACCAAAATC-3′ Reverse: 5′-TGACAATTTCATCCCGAACG-3′
*Nrf-2*	Forward: 5′-CAGTCAGCGACGGAAAGAGT-3′ Reverse: 5′-GGCTACCTGAGCAACAGAAG-3′
*p21*	Forward: 5′-CTGAGCCGCGACTGTGATGCG-3′ Reverse: 5′-GGTCTGCCGCCGTTTTCGACC-3′
*p53*	Forward: 5′-CTCTGACTGTACCACCATCCACTA-3′ Reverse: 5′-GAGTTCCAAGGCCTCATTCAGCTC-3′
*p62*	Forward: 5′-CCGTGAAGGCCTACCTTCTG-3′ Reverse: 5′-GCACTTGTAGCGGGTTCCTA-3′
*Snail*	Forward: 5′-ATGCCGCGCTCTTTCCTCGTCAGG-3′ Reverse: 5′-TCAGCGGGGACATCCTGAGCAGCC-3′

## Data Availability

Data associated with the publication are available upon request by the corresponding author.
